# Accounting for biological variation with linear mixed-effects modelling improves the quality of clinical metabolomics data

**DOI:** 10.1016/j.csbj.2019.04.009

**Published:** 2019-04-22

**Authors:** Kwanjeera Wanichthanarak, Saharuetai Jeamsripong, Natapol Pornputtapong, Sakda Khoomrung

**Affiliations:** a Department of Biochemistry and Siriraj Metabolomics and Phenomics Center, Faculty of Medicine Siriraj Hospital, Mahidol University, 2 Wanglang Road, Bangkok Noi, Bangkok 10700, Thailand; b Data Management and Statistical Analysis Center, Faculty of Public Health, Khon Kaen University, Khon Kaen 40002, Thailand; c Research Unit in Microbial Food Safety and Antimicrobial Resistance, Department of Veterinary Public Health, Faculty of Veterinary Science, Chulalongkorn University, 39 Henri-Dunant Road, Pathumwan, Bangkok 10330, Thailand; d Department of Biochemistry and Microbiology, Faculty of Pharmaceutical Sciences, Chulalongkorn University, Bangkok, 10330, Thailand; e Center of Excellence in Systems Biology, Faculty of Medicine, Chulalongkorn University, Bangkok 10330, Thailand; f Center for Innovation in Chemistry (PERCH-CIC), Faculty of Science, Mahidol University, Rama 6 Road, Bangkok 10400, Thailand

**Keywords:** Confounding biological factors, Linear mixed-effects models, Metabolomics, Multivariate analysis, Subject metadata

## Abstract

Metabolite profiles from biological samples suffer from both technical variations and subject-specific variants. To improve the quality of metabolomics data, conventional data processing methods can be employed to remove technical variations. These methods do not consider sources of subject variation as separate factors from biological factors of interest. This can be a significant issue when performing quantitative metabolomics in clinical trials or screening for a potential biomarker in early-stage disease, because changes in metabolism or a desired-metabolite signal are small compared to the total metabolite signals. As a result, inter-individual variability can interfere subsequent statistical analyses. Here, we propose an additional data processing step using linear mixed-effects modelling to readjust an individual metabolite signal prior to multivariate analyses. Published clinical metabolomics data was used to demonstrate and evaluate the proposed method. We observed a substantial reduction in variation of each metabolite signal after model fitting. A comparison with other strategies showed that our proposed method contributed to improved classification accuracy, precision, sensitivity and specificity. Moreover, we highlight the importance of patient metadata as it contains rich information of subject characteristics, which can be used to model and normalize metabolite abundances. The proposed method is available as an R package lmm2met.

## Graphical abstract

**Figure:**
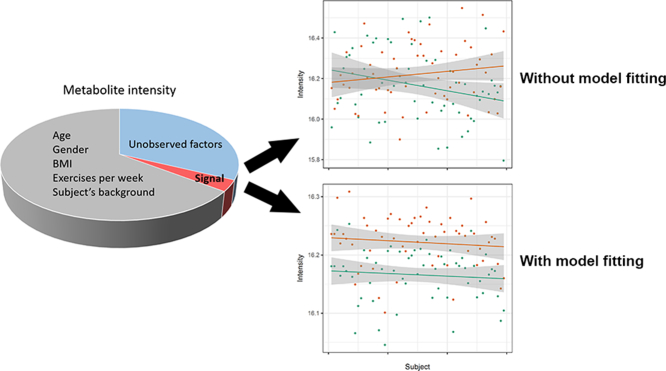

## 1 Introduction

Metabolites are the low molecular weight compounds that participate in the network of chemical reactions supporting cell growth and function [[1], [2], [3]]. It is now widely accepted that metabolomics or metabolite profiling can elucidate metabolic and physiological statuses of living systems in response to genetic conditions, pathological stimuli and environmental stress [4,5]. Metabolomics facilitates a deeper understanding of pathophysiological stages, mechanisms of diseases, and drug responses. Furthermore, the technology is becoming a valuable tool for the identification of more specific and sensitive diagnostic and prognostic disease biomarkers [6]. Thus, metabolomics is being exploited in several aspects of both modern or traditional biomedical research.

Metabolomics analysis aims to identify and quantify all possible metabolite species from bodily fluids (such as serum, plasma, saliva and urine) or tissue samples. The analysis focuses on small molecules with molecular weight <1 kDa [7]. With the recent advancements of both mass spectrometry (MS) and nuclear magnetic resonance (NMR), approximately 100–1000 different small molecules can be routinely detected and quantified. Nonetheless, metabolomics conducted in clinical research usually suffers from high dimensionality, small sample sizes, and low signal-to-noise ratios [8]. The consequences of such issues include the loss of statistical power, low confidence in biomarker identification, and poor reproducibility. Moreover, quantitative metabolomics in clinical trials or in the discovery of disease-onset biomarkers is highly challenging due to a small change in metabolism.

The analysis of metabolomics data is a considerable challenge because the intensity of a metabolite signal from MS or NMR is the combination of the desired signal from an analysis, undesired signals from inter-subject differences, and noise from technical analysis [9,10]. The key sources of signal contamination from technical analyses are personal errors, batch effects, fluctuation of instrumental sensitivity, changes of mobile-phase compositions, and column degradation or loss of chromatographic performance over time. To overcome these, a number of approaches have been proposed, including normalization by the median, the sum of squares, regression of abundances in each sample [11], the use of quality assurance/quality control through samples or internal standards [10,[12], [13], [14]] and standard reference materials [15,16]. Using these strategies to standardize metabolite abundances prior to univariate or multivariate analysis greatly enhances the quality of metabolomics data as well as biological interpretation. Although these approaches can largely remove undesired signals from the dataset, biological factors such as diet, medication, physical activity, genetic background and demographic diversity [9] remain a significant challenge in data analysis, and can be misleading regarding the outcome of phenotypic detection.

Linear mixed-effects models (LMMs) are considered robust and powerful tools for analyzing data with complex, correlated structures and multiple sources of variation [17]. An individual parameter in the LMM model represents either a fixed or a random effect. A fixed effect is an average effect of a particular experimental factor that is constant across an entire population [18]. A random effect is unobserved random variation associated with individual subject that is randomly selected from a population [18,19]. The method explicitly incorporates both fixed effects and random effects, which accounts for unwanted sources of variation in individual measurements. LMM fitting has had great successes in univariate analysis for the identification of genes or metabolites significantly affected by a factor of interest [[20], [21], [22], [23], [24]]. However, using an LMM approach for processing metabolomics data has not been thoroughly exploited. The method proposed by De Livera et al. applies the LMM method to remove undesired variation [9,10]. However, this approach requires nonchanging metabolites (metabolites that are not changed or affected by factors under study) of a particular experiment beforehand. The studies from Westerhuis et al. [25] and Liquet et al. [26] use a multilevel approach, comparable with the LMM method, in conjunction with partial least squares–discriminant analysis (PLS-DA) and sparse PLS-DA (sPLS-DA) to examine the treatment effect from repeated measures experiment. This approach separates between-subject variation from within-subject variation before applying the multivariate approaches to the within-subject variation matrix, which is assumed to represent the treatment effect [26]. However, sources of subject variation are not explicitly delineated. In contrast, the LMM approach enables subject metadata, which includes information such as demographics, medication status, food intake and other subject characteristics, to be fully utilized and flexibly incorporated to form LMMs.

In this study, we propose the use of LMMs as an additional data processing step for adjusting an individual metabolite signal in metabolomics data. We highlighted the core benefit of the LMM approach which allows the formulation of various random effect terms to cope with a variety of unobserved random variance. We demonstrated the benefit of the LMM approach using patient metadata to model a metabolomics data matrix. Predictive power of discriminative features selected from PLS-DA (and OPLS-DA) with and without the use of LMMs, and from multilevel PLS-DA, was evaluated and compared. All studies were performed using published clinical metabolomics data.

## 2 Materials and Methods

### 2.1 Datasets

Two published metabolomics datasets were used in this study to demonstrate and evaluate the utility of the proposed LMM method. The first study was that of Liesenfeld et al. [27], a dataset of adipose tissue samples from 59 colorectal carcinoma (CRC; including both colon and rectal cancer) patients with tumor stages ranging from I-IV. Two types of adipose tissues, subutaneous adipose tissue (SAT) and visceral adipose tissue (VAT), were dissected from the same patient. In this study, the authors report primary metabolites and lipids measured using gas chromatography time-of-flight mass spectrometry (GC-TOF MS) and liquid chromatography qTOF MS (UPLC-QTOF MS). The data is available through the Metabolomics Workbench [28] with assigned Project ID: PR000058; Study ID: ST000081. We used 158 annotated lipids from both VAT and SAT in 59 patients and the patients' metadata is summarized in Table 1. The second dataset was obtained from Wikoff et al. [21] where they performed metabolite profiling of matched malignant and non-malignant lung tissues from 39 early stage adenocarcinoma patients. The data is available through the Metabolomics Workbench [28] with assigned Project ID: PR000305; Study ID: ST000390. A total of 462 metabolites were measured by GC-TOF MS, 183 of which had annotated chemical structures. The metadata of this study are given in Table S-1, Table 1. Both datasets were pre-processed as described previously [21,27] and log2-transformed prior to use in this study.

### 2.2 Linear mixed-effects models

The standard formulation of an LMM^29^ is described in eq. 1:

(1) $$y_{\mathrm{ij}}=\beta_{0}+\mu_{0i}+\Sigma \beta_{\mathrm{jk}}X_{\mathrm{ik}}+\epsilon_{\mathrm{ij}}$$

**Table 1:** Metadata of patients (*n* = 59) with colorectal carcinoma.

Variables	Values	
Age, mean ± SD	62.92 ± 13.71	
Sex, n (%)		
Female	16 (27)	
Male	43 (73)	
BMI, mean ± SD	27.14 ± 4.38	
Tumor stage, n (%)		
Stage I	9 (15)	
Stage II	23 (39)	
Stage III	16 (27)	
Stage IV	11 (19)	
Tumor location, n (%)		
SAT: Colon	26 (44) 25 (42) 33 (56) 34 (58)	
SAT: Rectum VAT: Colon VAT: Rectum		

We denote the abundance of the *j*^*th*^ metabolite for the *i*^*th*^ sample as *y*_*ij*_; where *i* = 1, 2, … , *the number of samples* (*n*), *j* = 1, 2, … , *the number of metabolites* (*m*), *β*_0_ is the overall mean or baseline level of metabolite abundance across the whole population (intercept), *μ*_0*i*_ is the random effect of the *i*^*th*^ sample (or an individual's variation) and *ϵ*_*ij*_ is the random error of the model associated with samples and metabolites. The levels of metabolites are the consolidation of between-subject variation or unobserved random variables and the fixed effects, which account for the effect of the factor of interest (e.g. treatment) and the effects of other known endogenous factors (e.g. age and gender). These factors are represented as *X*_*k*_; where *k* = 1, 2, … , *the number of fixed effects* (*p*) and *β*_*k*_ is the coefficient or the level of effect associated with factor *X*_*k*_.

**Fig. 1:**
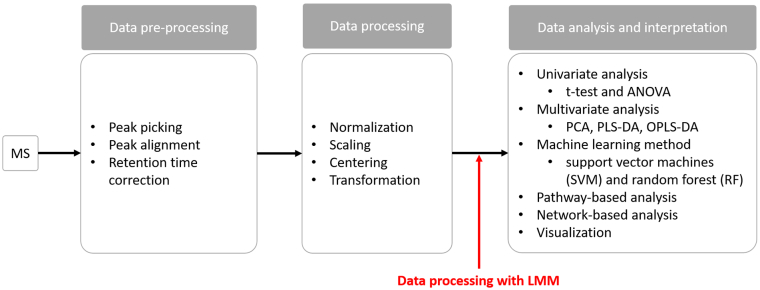
Metabolomics data analysis workflow. For the general analysis workflow, the main steps include data pre-processing, data processing, and statistical analysis and biological interpretation. Data pre-processing, such as noise reduction, peak matching and retention time correction, is to manage raw/original signals acquired from mass spectrometry (MS) platforms. Data processing includes normalization, scaling, centering and transformation. In this study, the LMM method (in red) is proposed as an additional step performed after the conventional data processing tasks. The processed data matrices are subsequently assessed to probe metabolite biomarkers discriminating between experimental conditions by a variety of statistical approaches, including univariate analysis, multivariate analysis and machine learning methods. The downstream analysis of the statistical outputs employs pathway-based analyses, network-based analyses and visualization tools to aid biological interpretation of the statistical results. (For interpretation of the references to colour in this figure legend, the reader is referred to the web version of this article.)

The workflow of metabolomics data analysis is illustrated in Fig. 1 [30]. For the proposed data processing step, each measured metabolite is fitted to an LMM. The effects of experimental factors and known subject variations are assumed to have a fixed effect on a population. Accordingly, both the factors of the study and subject characteristics explicitly form the fixed-effects part of the model. Meanwhile, other unobserved or uncontrolled random factors are included in the random effects. In general, the random-effect term can be formulated in different forms as described in Bates et al. [29]. The random effect is assigned depending on the grouping factor and the assumption of the study. In our context, the random-effect formulation uses the paired structure of the data to indicate a random intercept, where mean levels of metabolites are treated as different among individuals. This random-effect term is considered the simplest form [29] and was used in this study.

The LMM approach is flexible to formulate a variety of the random-effect terms specific for the experimental design and assumption of each study, such as single and multiple random-effect terms, nested, crossed, correlated and uncorrelated random effects [29]. Herein, we additionally investigated and compared different formulations of the random-effect terms to model metabolomics data matrices of adipose tissue and lung tissue samples. Detailed information and discussion regarding this investigation is included in Supplementary file S-1.

We developed the R package lmm2met to process a metabolomics matrix with the LMM approach. The package was implemented on a linear model-fitting procedure of the lme4 package [29]. The format of the random-effect term follows lme4 formulas [29]. The output from LMM fitting is a matrix of processed metabolite profiles, which accounts for the given fixed effects and the subject-specific random effects. This is to ensure that the metabolite signals of the factor under study are minimally confounded by individual variation. The resulting matrix can be used further in multivariate methods such as PLS-DA and OPLS-DA. Moreover, the coefficients of fixed effects together with statistical significances by chi-square test are returned. R package lmm2met, online documentation, and tutorial is available at https://github.com/kwanjeeraw/lmm2met.

### 2.3 Evaluation of class prediction performance

**Fig. 2:**
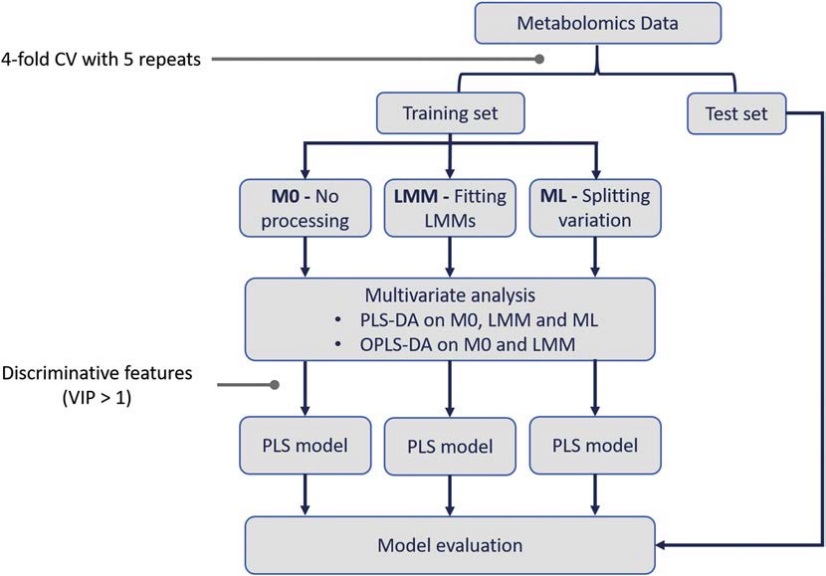
Flowchart of prediction evaluation. The original data (adipose tissue data or lung cancer data) was first partitioned into training and test sets with a repeated 4-fold cross validation (CV). This step was iterated five times to generate 20 groups of training and test data sets. There were three different approaches to process the training sets: M0, LMM and ML. PLS-DA was performed after M0, LMM and ML. OPLS-DA was performed only after M0 and LMM. Then, the discriminative features (VIP > 1) from each method were used to construct a PLS classification model and its prediction performance was evaluated by a corresponding test set.

A flowchart of the prediction evaluation process is given in Fig. 2. First, each dataset was partitioned into training and test sets with a repeated *k*-fold cross validation [31] (*k* = 4). This approximately gives 75% of the dataset as a training set and the remaining 25% as a testing set. Data partitioning was repeated five times resulting in 20 groups of training and test sets. We investigated differences between three data processing approaches as I) the original data with no extra processing (M0), II) the data fitted with an LMM approach (LMM), and III) the data processed with a multilevel-based approach (ML). The M0- and LMM-processed training sets were subsequently analyzed with PLS-DA and OPLS-DA. The ML-processed training sets were subjected to only PLS-DA as implemented in the mixOmics package. The discriminative features from each strategy were selected for training and building a PLS classification model, where its class prediction was assessed by the corresponding test set afterwards. The significant features were selected based on variable importance in projection score (VIP > 1), which represents the contribution of individual feature to group separation [32].

We measured and compared the performance metrics in terms of accuracy, precision, sensitivity and specificity among the different methods. The performance values of each PLS classification model are the average estimates of 20 subsets of training and test data. All analyses were performed using R (https://www.r-project.org/). The evaluation processes were carried out using the caret package [33]. The mixOmics package [34] was applied for PLS-DA and multilevel-based PLS-DA. OPLS-DA was performed with the ropls package [35]. Two published metabolomics datasets: adipose tissue data and lung cancer datasets were used to evaluate the proposed LMM approach.

## 3 Results and Discussion

### 3.1 Application of an LMM approach in analyzing adipose tissue data from colorectal cancer patients

We applied the LMM method, a flexible approach that incorporates metadata, to readjust the signal of metabolite intensity. This is an additional data processing step from conventional metabolomics data analysis (Fig. 1). We demonstrated the proposed method with lipid profiles of adipose tissue samples. The metadata information of 59 patients (Table 1) was used to formulate LMMs. From the original study [27,28], the authors predominantly investigated differences in metabolite compositions in VAT and SAT. We also assumed that the total signal of a metabolite measured experimentally may be influenced by intra-subject factors or subject characteristics [36]. Hence, we considered tissue type, age, BMI, sex, tumor location and tumor stage as the fixed effects. The coefficients of these factors were estimated during LMM fitting (Table S-2). Patient identifier (Id) was assigned as a random-effect term in the model, assuming mean levels of metabolite abundances are varied among patients because of other unobserved factors. This strategy is particularly useful for explicit adjustment of multiple sources of biological factors. It is more flexible than other methods such as the ML method which exclusively considers subject characteristics as a random subject effect [25,26].

**Fig. 3:**
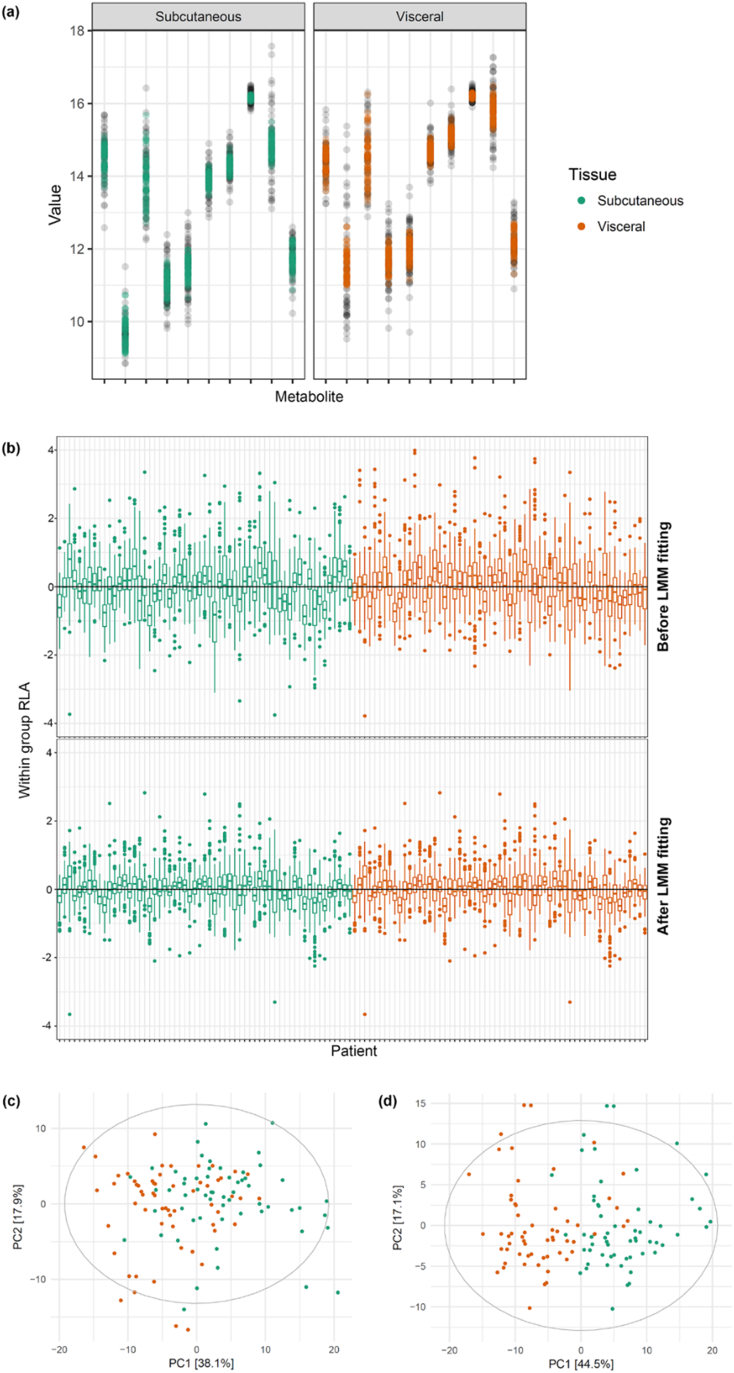
Effects of LMM fitting represented by various plots. (a) Variance of metabolites from both adipose tissues, before (black) and after (colored) LMM fitting. (b) Within-group RLA plots. (c) PCA score plots of adipose tissue samples before and (d) after LMM fitting.

Initially, we investigated how the additional data processing with LMM fitting affects metabolite abundances. It can be seen that variance of a metabolite across all patients was substantially reduced and outliers were corrected after LMM fitting (Fig. 3a and Figure S-1, Fig. 1). The use of within group relative log abundance (within group RLA) [9] plots is to examine overall variation of patients. These plots were obtained by first computing the median of each metabolite within each tissue type, then subtracting the median from each metabolite within groups and illustrating as subject-wise boxplots [9,10]. Here, LMM fitting helped minimize patient variation as the subject-wise boxplots after LMM fitting had a median closer to zero and showed less variability within tissue type (Fig. 3b). To evaluate the performance of LMM fitting, we first employed principle component analysis (PCA) to observe intrinsic variability structure of the data before and after LMM fitting. The PCA score plot of data fitted with LMM (Fig. 3d) depicted better separation between SAT and VAT with 6% increase in explanation of variation on PC1 in comparison with the original data (Fig. 3c). These results suggested that accounting for both fixed and random effects to refine a metabolite signal could clearly improve the explanation of variance on PC1 generally capturing the maximum variation of the data.

**Fig. 4:**
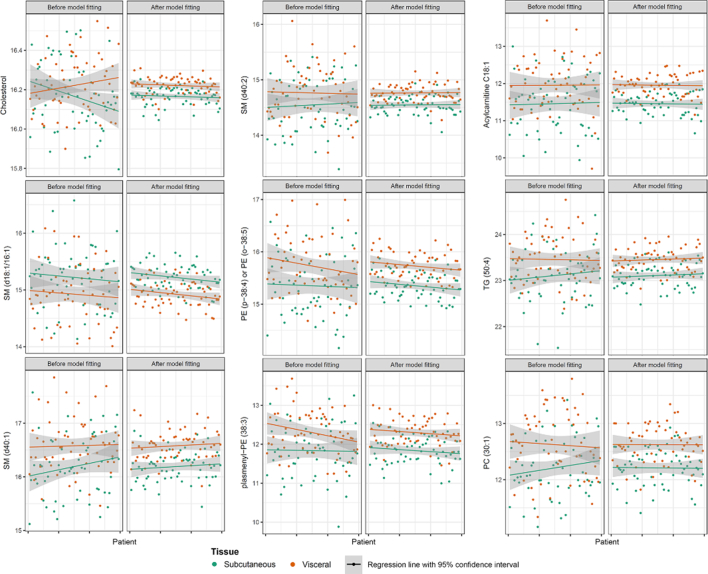
Variation of nine significant metabolites exclusively identified in the LMM dataset before and after LMM fitting. Changes of VIP scores after LMM fitting (ΔVIP): Cholesterol; ΔVIP = 0.52, SM (d40:2); ΔVIP = 0.43, acylcarnitine; ΔVIP = 0.32, SM (d18:1/16:1); ΔVIP = 0.28, PE (p-38:4) or PE (o-38:5); ΔVIP = 0.25, TG (50:4); ΔVIP = 0.20, SM (d40:1) ΔVIP = 0.11, plasmenyl-PE (38:3) ΔVIP = 0.06, PC (30:1) ΔVIP = 0.02.

Next, we performed PLS-DA on the LMM and M0 datasets. In total, there were 69 and 66 significant metabolites or discriminative metabolites (VIP > 1) that were identified in the LMM and M0 datasets, respectively, in which 60 metabolites were commonly identified in all processed datasets (Figure S-1, Fig. 2 and Table S-3). Nine unique metabolites were captured in the LMM dataset, whereas there were six distinct metabolites from the M0 dataset. Fig. 4 presents an overview structure of those metabolites solely identified in the LMM dataset. Within the same tissue, metabolite variance was clearly minimized, such that the pattern of a metabolite between two tissues became more distinct after LMM fitting.

In addition, we investigated the changes in VIP scores of those unique metabolites from the LMM and M0 datasets (Figure S-1, Fig. 3). Among them, the VIP score of cholesterol showed the largest increases from the original signal (0.52 arbitrary units higher). The VIP scores of SM (d40:2) and Acylcarnitine C18:1 were the second and the third largest, respectively. Before LMM fitting, cholesterol signals were randomly mixed and indistinguishable (Fig. 4, Cholesterol). One could assume that cholesterol was neither different between the tissue types nor obscured by other factors, and was therefore not identified by PLS-DA in the M0 dataset. Moreover, cholesterol was not recognized as significantly different between two tissues from the ML dataset following by PLS-DA (ML-PLS-DA). Though ML approach is a data processing step to cope with confounding variables, this approach does not explicitly decompose sources of subject variations like the LMM approach. Conversely, after processing data through LMM, differences in cholesterol signals between SAT and VAT were enhanced, in which the cholesterol in VAT was more abundance than SAT (coefficient of VAT = 0.05 and *p*-value = .05, Table S-2).

In the original study, Liesenfeld et al. (2015) observed higher inflammatory signaling along with free-arachidonic acid levels in VAT than in SAT [27]. Arachidonic acid has been previously shown to regulate cholesterol metabolism [37]. Furthermore, it has been reported that metabolic dysfunction of lipid metabolism influences hormonal pathways, inflammation, and tumor progression [27,38]. High serum cholesterol levels have been associated with CRC [[38], [39], [40]] and other cancers such as prostate cancer [41] and breast cancer [42]. Wang et al. (2017) recently proposes that the activation of reactive oxygen species (ROS) and the mitogen-activated protein kinase (MAPK) pathway by low-density lipoprotein cholesterol (LDL) can elevate intestinal inflammation and CRC progression [39]. Thus, cholesterol is highly likely to play an important role in CRC development. As the cholesterol signal was found to significantly discriminate between VAT and SAT in our method, this suggested that the systematic adjustment of mixed effects in each metabolite could indeed increase sensitivity and a chance to identify important metabolites.

On the other hand, four metabolites of triglyceride (TG) and two metabolites of phosphatidylcholine (PC) lipid families were exclusively identified in the M0 dataset (Table S-3). The VIP scores of those metabolites slightly decreased after LMM fitting, however these scores varied between 0.92 and 1.00, which were still considerably high (Figure S-1, Fig. 3).

### 3.2 Metabolite concentrations relevant to endogenous factors

After performing PLS-DA on the LMM of the adipose tissue data, there were nine unique metabolites (cholesterol; sphingomyelin (SM) species; acylcarnitine; phosphatidylethanolamine (PE) species; triacylglycerol (TG) species and phosphatidylcholine (PC) species) captured in the LMM dataset (Fig. 4). The intra-group variance was minimized whereas inter-group variation became more distinct after LMM fitting to manage confounding variables. As reported by numerous studies, differences in physiological conditions such as age, gender and BMI were found to have influences on metabolite levels [[43], [44], [45]]. In addition to disease states, prescribed medication and environmental factors, the study from Thevenot et al. (2015) [43] identified that the concentrations of 108 urine metabolites of various classes (such as amino acids, organic acids, acylcarnitines, steroids and lipids) were correlated with age, BMI or gender. Correlations of age [46,47], BMI [48] and gender [49] to several lipid species (e.g. PC, PE and SM) are also present in several plasma and serum lipid profiling studies. It can be seen that between-subject variations of metabolite levels are expected in clinical metabolomics. Therefore, data processing such as the LMM method is needed to eliminate other sources of unrelated variation.

### 3.3 Comparing prediction performance between methods

We measured classification accuracy, precision, sensitivity and specificity to compare performance of the LMM, M0 and ML methods. Accuracy indicates overall aspects of the test performance. Precision represents the proportion of positive predictions that are correct, so high precision can mean a lower number of false positives. Sensitivity reveals how many actual positive cases can be detected, also implying the number of false negatives. Specificity is the proportion of actual negative cases correctly returned.

**Fig. 5:**
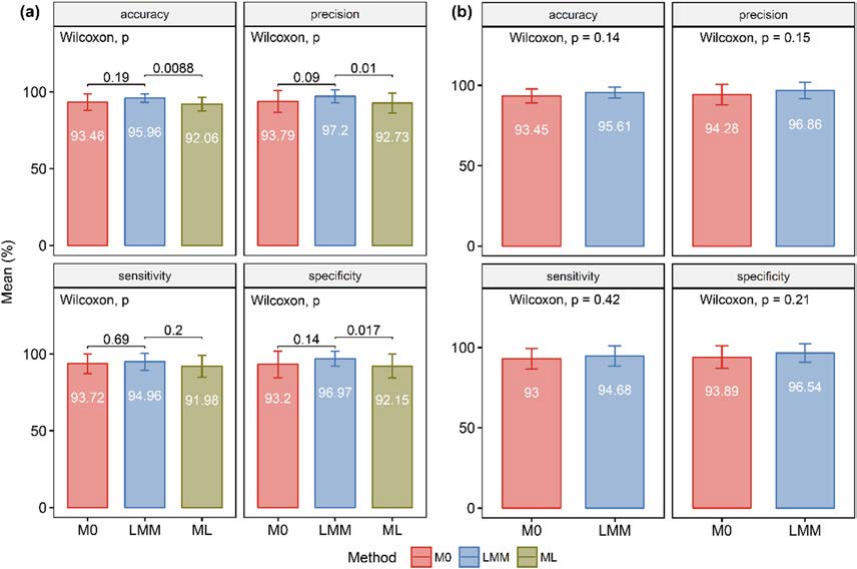
Comparison of prediction performance using adipose tissue metabolomics data. (a) Performance metrics of PLS models built from LMM-, M0- and ML- PLS-DA and (b) from LMM- and M0-OPLS-DA. Wilcoxon signed-rank tests were performed to compare the LMM method to the M0 and the ML method. *P*-values are displayed and the level of significance was set at *p* < .05. Mean values of the performance metrics are shown in each bar with error bars as standard deviations.

For adipose tissue data, all three data processing approaches contributed to considerably high prediction performance with an average value higher than 90% (Fig. 5). This is because the dataset is from obviously histologically different tissue types. Though, for all performance metrics, the proposed LMM method exhibited an improvement over the M0 and ML methods. Surprisingly, the M0-PLS-DA also performed better than the ML-PLS-DA in all prediction performance aspects. For PLS-DA on the LMM, M0 and ML -processed datasets, accuracy, precision and specificity were significantly different between the LMM and ML method (*p* < .05). Mean differences between the LMM and M0 methods and the LMM and ML methods were about 1%–4%. Large differences between mean precision and mean specificity of the LMM compared to the M0 and ML methods were observed (approximately 4%), indicating that properly accounting for subject variation could assist in reducing the number of false positives or noise. Regarding OPLS-DA on the LMM and M0 -processed datasets, though there was no significant difference between the methods, the proposed LMM method showed better performance over the M0 method. Standard deviations of the M0 and ML methods were higher than those of the LMM method. This implies that the prediction performance of the M0 and ML method varies between subsets of training and test data (i.e. some subjects are not well representative of the target population), affirming the overfitting issue of PLS-DA and OPLS-DA [1]. Therefore, an additional processing step is needed, as it can return a more robust signal as a result of systematic adjustment of multiple sources of variation.

We also examined the performance of all three methods on the GC-TOF MS metabolomics data. The original study observed metabolic differences in malignant and matched non-malignant lung tissues [21]. Prediction performance of the M0, LMM and ML approach was approximately 80% (Figure S-1, Fig. 4). Overall, the performance metrics of the proposed LMM method were higher than the M0 and ML methods (except specificity, for which the ML method slightly outperformed the LMM method), though there was no statistical significance in these differences. The largest differences observed were those between mean sensitivity of the LMM and M0, and between LMM and ML, which were approximately 2% and 1.6%, respectively. This suggested that our proposed approach improves the ability to discover putative discriminants after individual variation adjustment.

## 4 Conclusion

The identification of small, yet important, signals among a large pool of confounding sample variation is a considerable challenge in clinical metabolomics. Several factors such as technical variance and the prevalence of uncontrolled variables such as individual differences in race, gender, age, diet, and lifestyle, highly influence phenotypic detection. Appropriate procedures are needed to eliminate or normalize technical and other sources of unrelated variation. In this study, we proposed an additional data processing step which uses the LMM approach to accommodate variation in features of interest and individual variances, and to promote identification of discriminative metabolites in subsequent multivariate analyses.

We demonstrated the benefit of the proposed method with published clinical metabolomics data. In brief, subject-specific variation in each metabolite was systematically minimized. The LMM fitting followed by PLS-DA or OPLS-DA improved overall prediction performance of PLS classification models. There were more discriminative features reported from LMM-PLS-DA than standard PLS-DA, suggesting that an additional data processing step increases sensitivity, albeit with the potential introduction of false positives. Therefore, further experiments are recommended to validate the results. However, at current stage, our proposed method exhibited superior performance, as it demonstrated superior accuracy, precision, sensitivity and specificity than the M0 and ML methods in both UPLC-QTOF MS and GC-TOF MS datasets (except specificity of GC-TOF MS dataset, for which the ML method was slightly better than the LMM method). The use of subject metadata combined with metabolomics data is increasingly being incorporated into metabolomics data analysis [28]. The proposed method enables the full and flexible utilization of subject metadata. It can be considered as an additional data processing module in the metabolomics data analysis pipeline.

The following are the supplementary data related to this article.

Table S-1 Metadata of patients (n = 39) with early stage lung adenocarcinoma. Table S-1

Table S-2 Coefficients of fixed effects and statistical significant levels by chi-square test. Table S-2

Table S-3 List of significant metabolites and VIP scores from the LMM- and M0-PLS-DA of CRC samples. Table S-3

Supplementary file S-1 Investigation of different random-effect terms. Supplementary file S-1

Figure S-1 Boxplot of metabolite signals before and after LMM fitting. Figure S-1

Figure S-2 Venn diagram of significant metabolites from the LMM- and M0-PLS-DA of CRC samples Figure S-2

Figure S-3 VIP scores of significant metabolites uniquely identified in the LMM and M0 datasets Figure S-3

Figure S-4 Comparison of prediction performance using lung tissue metabolomics data. Performance metrics of PLS models built (a) from LMM-, M0- and ML- PLS-DA and (b) from LMM- and M0-OPLS-DA. Wilcoxon signed-rank test was performed to compare between the LMM to M0 and to ML method. P-values are displayed (p) and significant level is p<0.05. Mean values of the performance metrics are shown in each bar with error bars as standard deviations Figure S-4
