# Supplementary material for: Accounting for biological variation with linear mixed-effects modelling improves the quality of clinical metabolomics data
Source: Comput Struct Biotechnol J. 2019 Apr 22;17:611–8. doi: 10.1016/j.csbj.2019.04.009 (PMC6506811; doi:10.1016/j.csbj.2019.04.009)
Supplement: Table S-1 — Metadata of patients (n = 39) with early stage lung adenocarcinoma. [file mmc1.docx]

**Table S-1. Metadata of patients (n = 39) with early stage lung adenocarcinoma**

| Variables | Values |
| --- | --- |
| Age, mean ± SD | 72.33 ± 8.78 |
| Sex, n (%) |  |
| Female | 24 (62) |
| Male | 15 (38) |
| Pack per year, mean ± SD | 36.79 ± 25.40 |
