# Supplementary material for: Accounting for biological variation with linear mixed-effects modelling improves the quality of clinical metabolomics data
Source: Comput Struct Biotechnol J. 2019 Apr 22;17:611–8. doi: 10.1016/j.csbj.2019.04.009 (PMC6506811; doi:10.1016/j.csbj.2019.04.009)
Supplement: Supplementary file S-1 — Investigation of different random-effect terms. [file mmc4.pdf]

Investigation of different random-effect terms

With the LMM approach, it is flexible to formulate a variety of the random-effect terms specifically for the experimental design and assumption of each study, such as single and multiple random-effect terms, nested, crossed, correlated and uncorrelated random effects[27]. Herein, we examined and compared different formulations of the random-effect terms to model both metabolomics data matrices (adipose and lung samples) in order to explore how different random-effect formulas impact on overall performance of the proposed data processing method. We formulated 3 different LMMs for adipose tissue data and for lung tissue data. The LMM formulas are given in the Table S1. The LMMs were conceived for performance analysis. These were not aimed for biological findings.

Table S1. Linear mixed-effects model formulas used in this study

Model	Fixed effects	Random-effect formula	Description	Data set
A1	Sex, Age, BMI, Stage, Location, Tissue	(1|Id)	Random intercept with fixed mean for each subject	Adipose samples
A2		(1|Id) + BMI|Tissue	Correlation between the slope for BMI and the intercept for each subject	
A3		(1|Id) + BMI||Tissue	No correlation between the slope for BMI and the intercept for each tissue type	
L1	Sex, Age, PackYear, Status	(1|SubjectID)	Correlation between the slope for BMI and the intercept for each tissue type	Lung samples
L2		(1|SubjectID) + (PackYear|Status)	Correlation between the slope for Pack per year and the intercept for each cancer status	
L3		(1|SubjectID) + (PackYear||Status)	No correlation between the slope for Pack per year and the intercept for each cancer status	

It can be seen that the PCA score plot of data fitted with any of the LMMs showed better separation between adipose tissue types (or lung cancer status) than the original data (Figure S1-S2). Prediction performance indices (accuracy, precision, sensitivity and specificity) among different LMMs was not significantly different (Figure S3). They were approximately 95% using adipose tissue metabolomics data and it were about 80% using lung tissue data sets. Comparing prediction performance between the different LMMs and other data processing approaches (M0 and ML method) also consistently shows that the proposed LMM method exhibited an improvement over the M0 and ML method for all performance metrics (Figure S4 and Figure S5). These results indicated that, regardless of random-effect formulas used, the proposed data processing method with the LMM approach could improve the explanation of variance in metabolomics data.

Figure S1. Effects of LMM fitting to adipose tissue samples represented by PCA score plots, (a) before LMM fitting; (b) after A1 model; (c) A2 model; and (d) A3 model fitting.

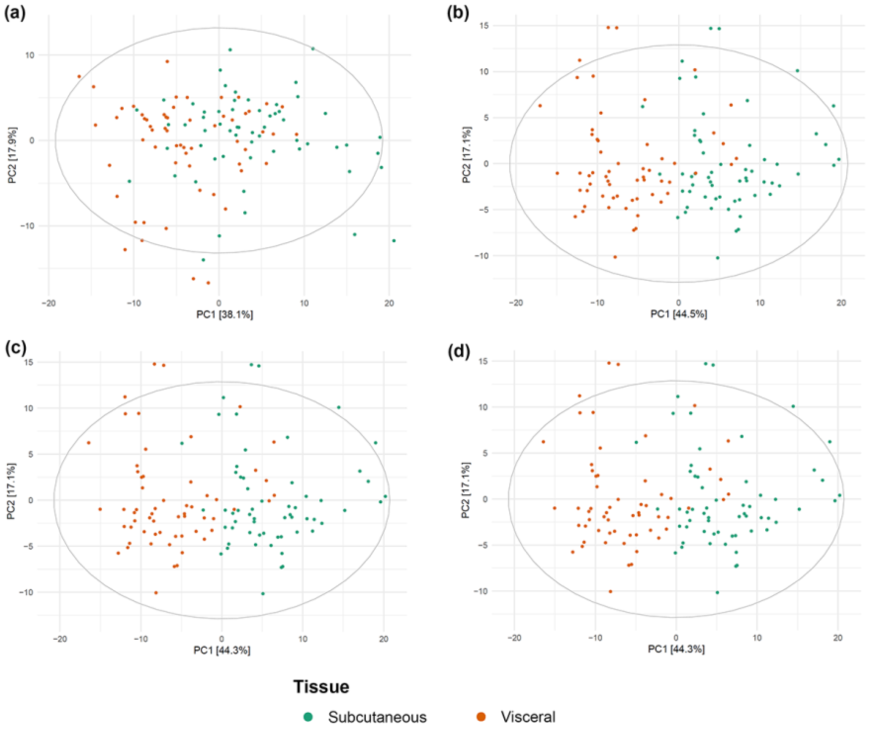

Figure S2. Effects of LMM fitting to lung tissue samples represented by PCA score plots, (a) before LMM fitting; (b) after L1 model; (c) L2 model; and (d) L3 model fitting.

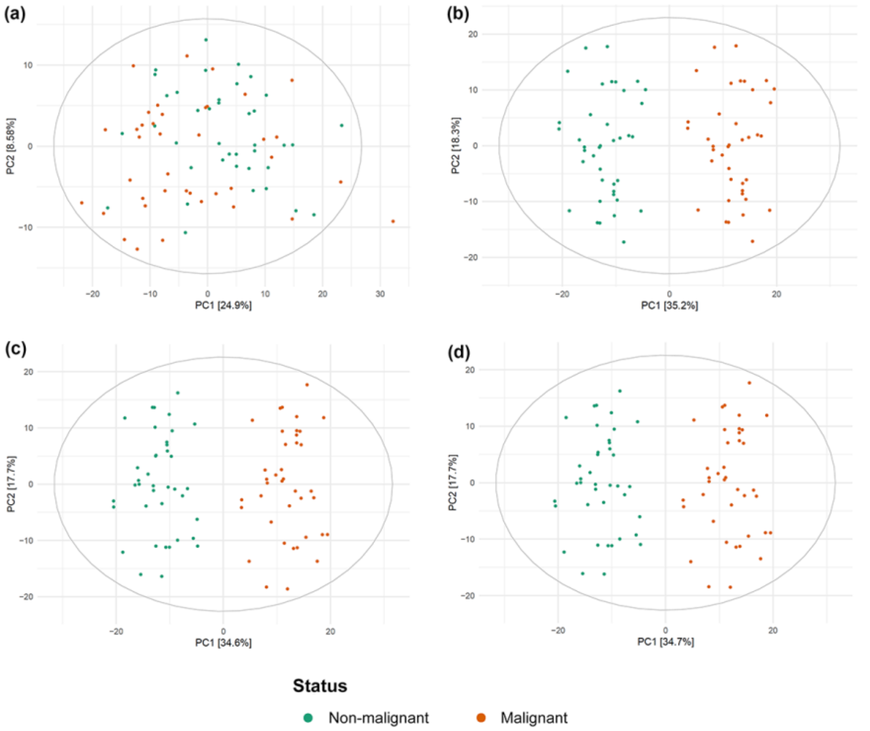

Figure S3. Comparison of prediction performance among the different LMMs. With adipose tissue metabolomics data, (a) performance metrics of PLS models built from A1-, A2- and A3- PLS-DA and (b) from A1-, A2- and A3- OPLS-DA. With lung tissue metabolomics data, (a) performance metrics of PLS models built from L1-, L2- and L3- PLS-DA and (b) from L1-, L2- and L3- OPLS-DA. A Kruskal–Wallis test was performed to compare among the LMMs. P-values are displayed and significance level was set at $p<0.05$. Mean values of the performance metrics are shown in each bar with error bars as standard deviations.

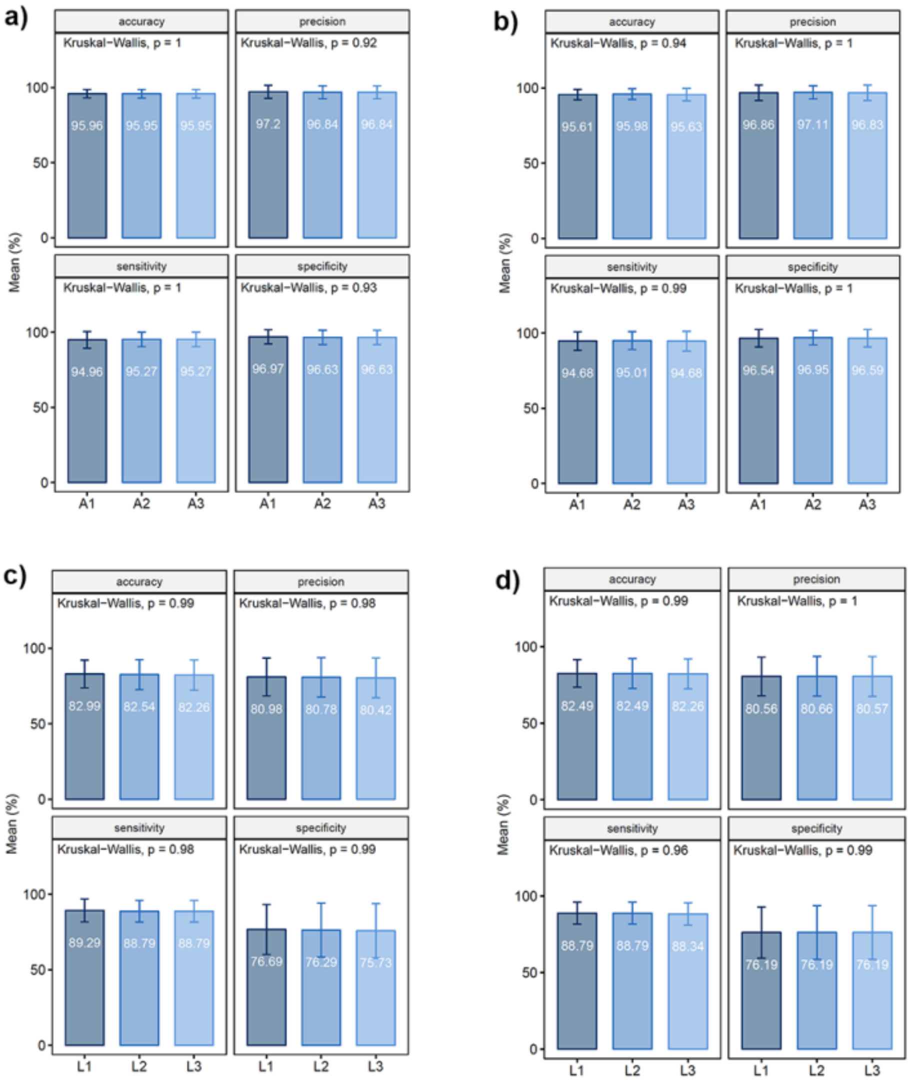

Figure S4. Comparison of prediction performance using adipose tissue metabolomics data. (a) Performance metrics of PLS models built from (a) A1-; or (b) A2; or (c) A3, M0- and ML- PLS-DA (left) and - OPLS-DA (right). Wilcoxon signed-rank test was performed to compare between the LMMs to M0 and to ML method. P-values are displayed and significance level was set at $p<0.05$. Mean values of the performance metrics are shown in each bar with error bars as standard deviations.

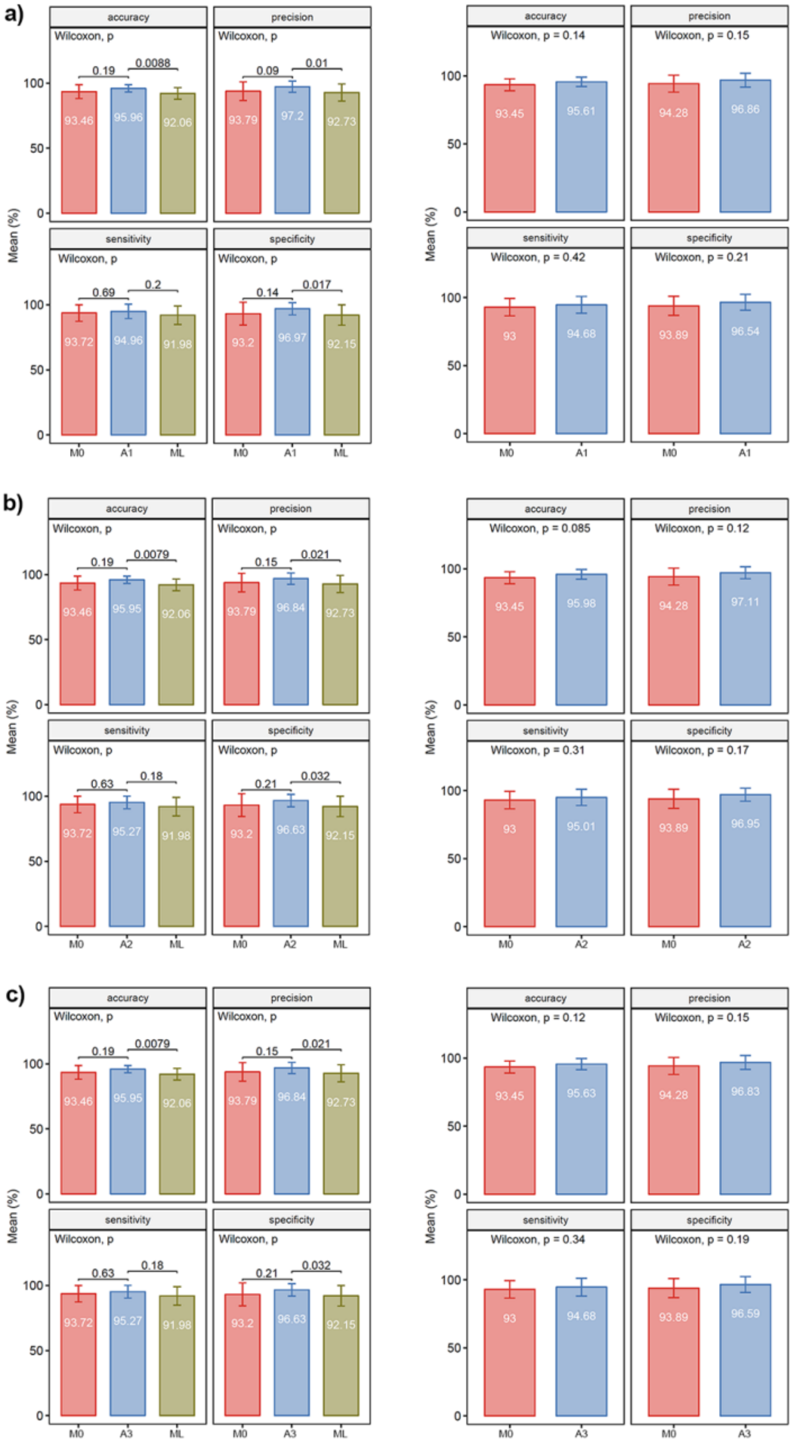

Figure S5. Comparison of prediction performance using lung tissue metabolomics data. (a) Performance metrics of PLS models built from (a) L1-; or (b) L2; or (c) L3, M0- and ML- PLS-DA (left) and - OPLS-DA (right). Wilcoxon signed-rank test was performed to compare between the LMMs to M0 and to ML method. P-values are displayed and significance level was set at p<0.05. Mean values of the performance metrics are shown in each bar with error bars as standard deviations.

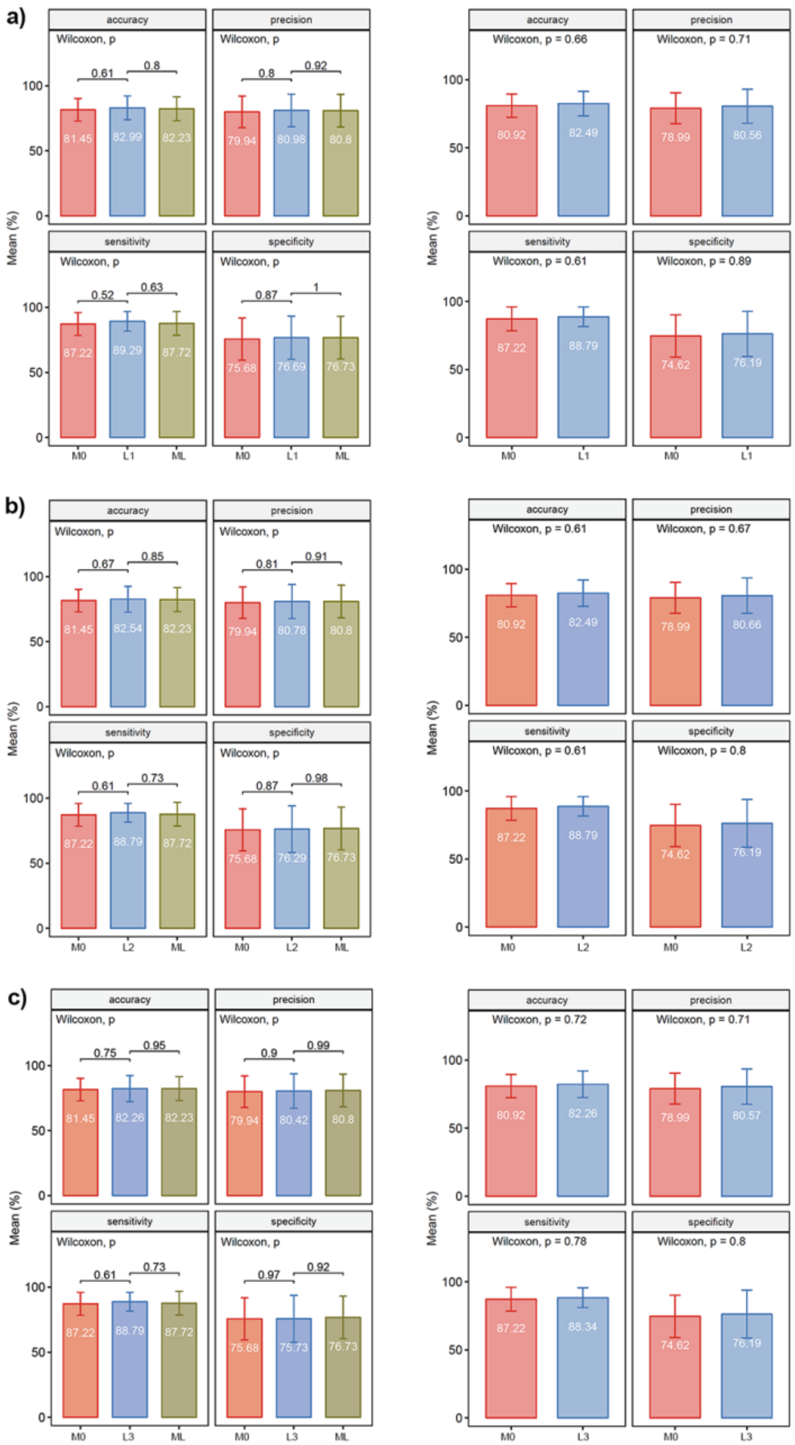

In addition, we examined the execution duration of the model fitting function to the 6 LMMs using a regular notebook, Intel Core i7 1.80 GHz, 8 GB Ram, 64-bit Windows 10 operating system. The results indicate that the time taken to execute the function is depended heavily on model complexity and the number of metabolite features (Table S2).

Table S2. Execution duration of the model fitting function

Model	Number of metabolites	Timing (second)
A1	158	25.28
A2		191.46
A3		44.12
L1	462	54.38
L2		254.70
L3		86.52
