# Supplementary figures and images for: Accounting for biological variation with linear mixed-effects modelling improves the quality of clinical metabolomics data
Source: Comput Struct Biotechnol J. 2019 Apr 22;17:611–8. doi: 10.1016/j.csbj.2019.04.009 (PMC6506811; doi:10.1016/j.csbj.2019.04.009)

**
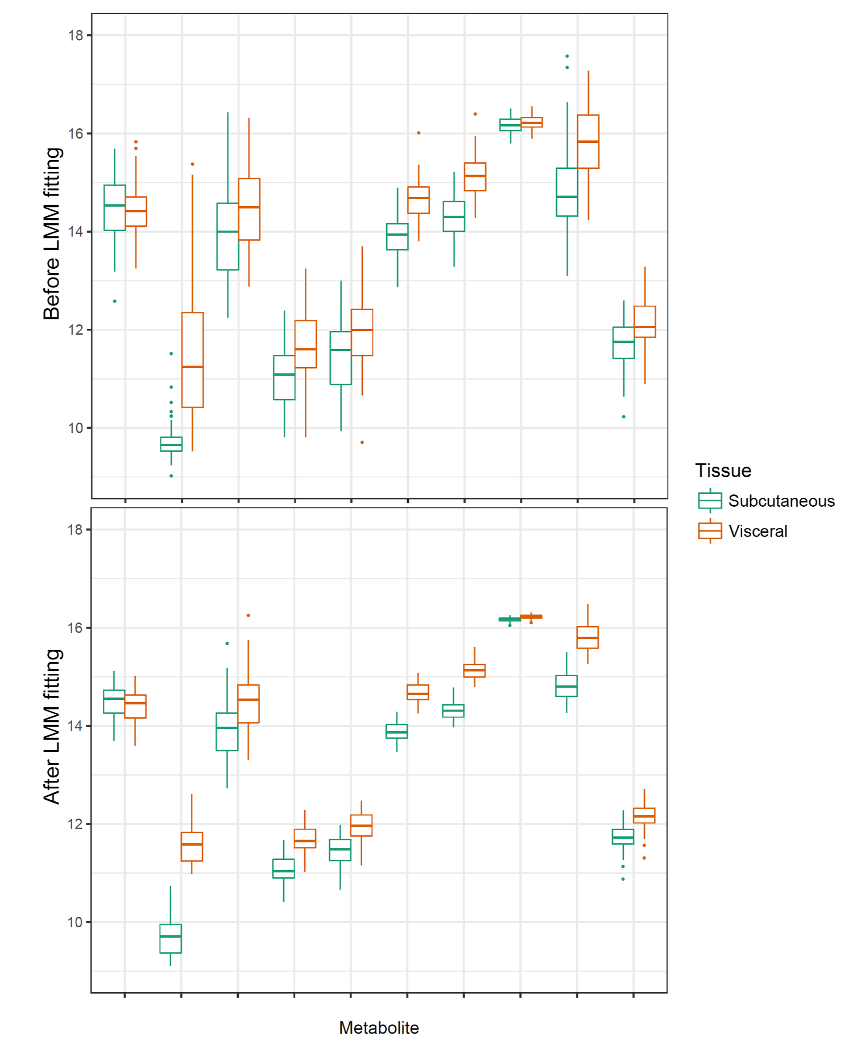
**

**Figure S-1.** Boxplot of metabolite signals before and after LMM fitting.

Supplement: Figure S-1 — Boxplot of metabolite signals before and after LMM fitting. [file mmc5.docx]

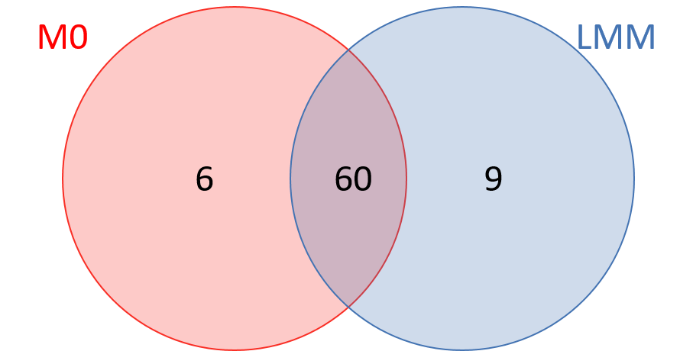


**Figure S-2.** Venn diagram of significant metabolitesfrom the LMM- and M0-PLS-DA of CRC samples.

Supplement: Figure S-2 — Venn diagram of significant metabolites from the LMM- and M0-PLS-DA of CRC samples [file mmc6.docx]

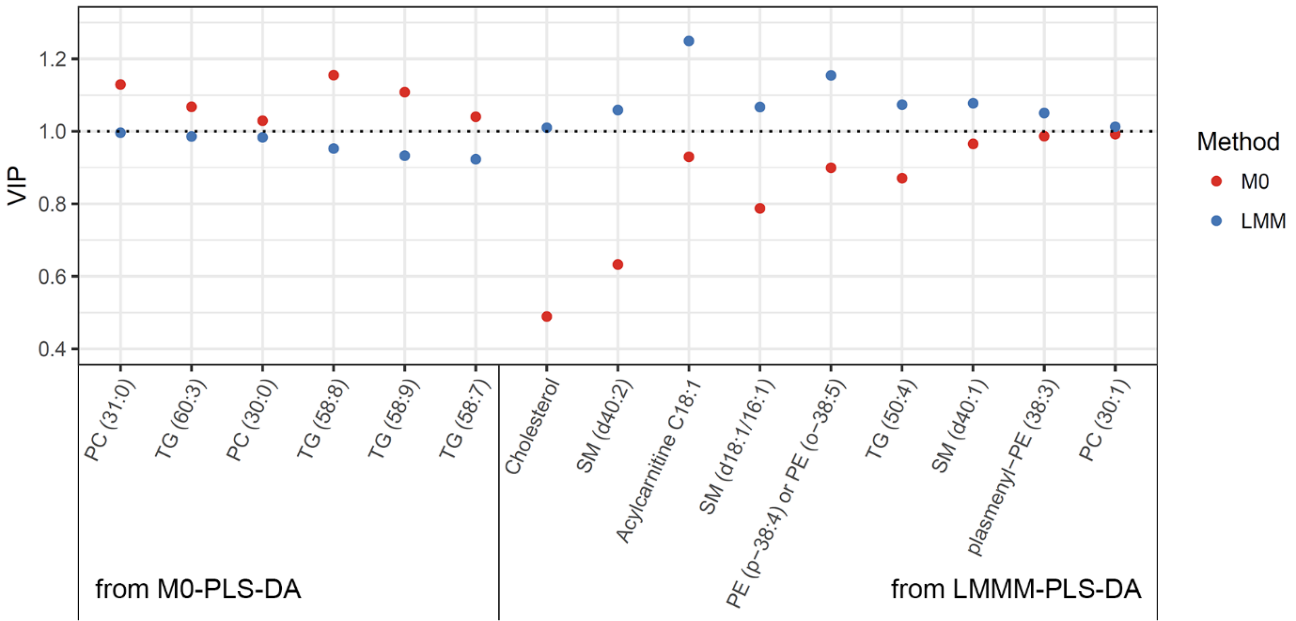


**Figure S-3.** VIP scores of significant metabolites uniquely identified in the LMM and M0 datasets.

Supplement: Figure S-3 — VIP scores of significant metabolites uniquely identified in the LMM and M0 datasets [file mmc7.docx]
