# Supplementary material for: Accounting for biological variation with linear mixed-effects modelling improves the quality of clinical metabolomics data
Source: Comput Struct Biotechnol J. 2019 Apr 22;17:611–8. doi: 10.1016/j.csbj.2019.04.009 (PMC6506811; doi:10.1016/j.csbj.2019.04.009)
Supplement: Figure S-4 — Comparison of prediction performance using lung tissue metabolomics data. Performance metrics of PLS models built (a) from LMM-, M0- and ML- PLS-DA and (b) from LMM- and M0-OPLS-DA. Wilcoxon signed-rank test was performed to compare between the LMM to M0 and to ML method. P-values are displayed (p) and significant level is p<0.05. Mean values of the performance metrics are shown in each bar with error bars as standard deviations [file mmc8.docx]

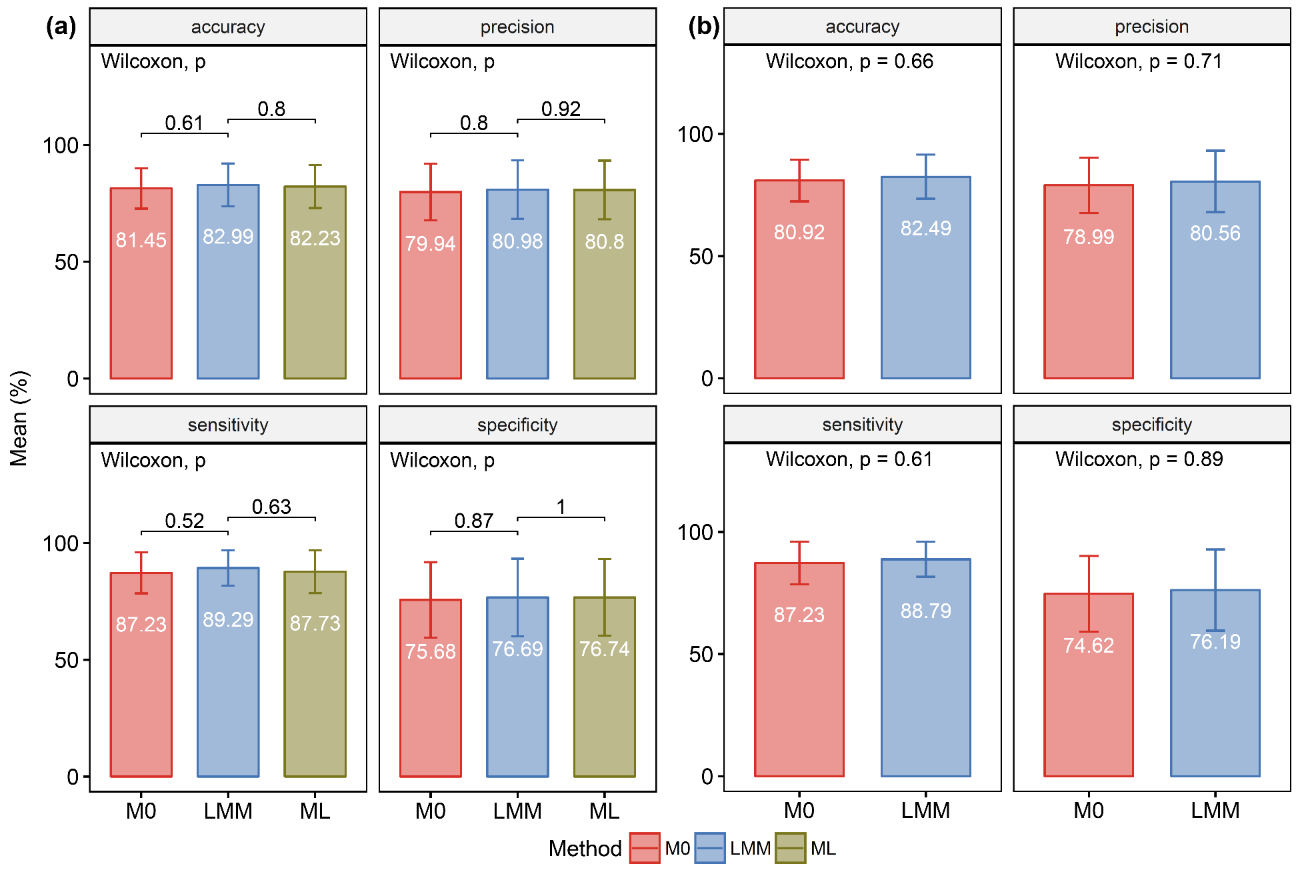


**Figure S-4.** Comparison of prediction performance using lung tissue metabolomics data. Performance metrics of PLS models built (a) from LMM-, M0- and ML- PLS-DA and (b) from LMM- and M0-OPLS-DA. Wilcoxon signed-rank tests were performed to compare the LMM method to the M0 and ML method. P-values are displayed and the level of significance level was set as p<0.05. Mean values of the performance metrics are shown in each bar with error bars as standard deviations.
